# The cytoprotective drug amifostine modifies both expression and activity of the pro-angiogenic factor VEGF-A

**DOI:** 10.1186/1741-7015-8-19

**Published:** 2010-03-24

**Authors:** S Dedieu, X Canron, HR Rezvani, M Bouchecareilh, F Mazurier, R Sinisi, M Zanda, M Moenner, A Bikfalvi, S North

**Affiliations:** 1 Inserm, U920, Talence, F-33400, France; 2 University of Bordeaux 1, Talence, F-33400, France; 3 Inserm, U 876, Bordeaux, F33000, France; 4 University of Bordeaux 2, Bordeaux, F-33000, France; 5 CNR, Milano, I-20131, Italy

## Abstract

**Background:**

Amifostine (WR-2721, delivered as Ethyol^®^) is a phosphorylated aminothiol compound clinically used in addition to cis-platinum to reduce the toxic side effects of therapeutic treatment on normal cells without reducing their efficacy on tumour cells. Its mechanism of action is attributed to the free radical scavenging properties of its active dephosphorylated metabolite WR-1065. However, amifostine has also been described as a potent hypoxia-mimetic compound and as a strong p53 inducer; both effects are known to potently modulate vascular endothelial growth factor (VEGF-A) expression. The angiogenic properties of this drug have not been clearly defined.

**Methods:**

Cancer cell lines and endothelial cells were used in culture and treated with Amifostine in order to study (i) the expression of angiogenesis related genes and proteins and (ii) the effects of the drug on VEGF-A induced *in vitro *angiogenesis.

**Results:**

We demonstrated that the treatment of several human cancer cell lines with therapeutical doses of WR-1065 led to a strong induction of different VEGF-A mRNA isoforms independently of HIF-1α. VEGF-A induction by WR-1065 depends on the activation of the eIF2alpha/ATF4 pathway. This up-regulation of VEGF-A mRNA was accompanied by an increased secretion of VEGF-A proteins fully active in stimulating vascular endothelial cells (EC). Nevertheless, direct treatment of EC with amifostine impaired their ability to respond to exogenous VEGF-A, an effect that correlated to the down-regulation of VEGFR-2 expression, to the reduction in cell surface binding of VEGF-A and to the decreased phosphorylation of the downstream p42/44 kinases.

**Conclusions:**

Taken together, our results indicate that amifostine treatment modulates tumour angiogenesis by two apparently opposite mechanisms - the increased VEGF-A expression by tumour cells and the inhibition of EC capacity to respond to VEGF-A stimulation.

## Background

First developed for its cytoprotective properties against radiation, amifostine is now approved by the US Food and Drug Administration for clinical use as a cytoprotector in several anti-cancer therapies [1]. Amifostine, also called Ethyol^®^, is a phosphorylated aminothiol (WR-2721). Its intracellular activity relies on its dephosphorylation by membrane bound alkaline phosphatase [2], thus producing the free thiol WR-1065. WR-1065 acts as a free radical scavenger and is considered to be the effective cytoprotector, affording protection against the toxic side effects of both chemotherapeutic agents and ionizing radiations [3-5]. This cytoprotection has been shown to be mostly effective on normal cells and does not interfere with the efficacy of anticancer treatment in tumours [6]. This selective effect on normal cells was attributed, in part, to the acidic microenvironment found in numerous tumour tissues, which decreases the rate of prodrug activation by the alkaline phosphatase and also to a lower expression of the enzyme in the tumour endothelium [2]. Abnormal tumour vasculature is also thought to lower the drug's access to tumour tissues [4]. Nevertheless, the drug does penetrate in cancer cells, even at a lower rate, and has often been described as enhancing the antitumour effects of chemotherapeutic agents and ionizing radiations [4,7,8]. This latter property, albeit not clearly understood, may depend on the activation of key regulatory proteins in cancer cells, such as the tumour-suppressive p53 protein, which is activated by amifostine and can block cell proliferation in p53 WT tumour cells, or enhance apoptosis due to chemotherapeutic agents in p53 deficient-tumour cells [9-12]. Amifostine has also been reported to act as a hypoxia-mimetic compound able to promote hypoxia-inducible factor (HIF)-1α accumulation both *in vitro *and *in vivo *[13]. HIF-1α is a major stress regulator induced by cancer cells in response to ischaemia during tumour development. Stabilized by hypoxia, HIF-1α heterodimerizes with the HIF-1β subunit and activates the transcription of different sets of genes involved in numerous cellular processes including metabolism, apoptosis and angiogenesis [14]. The most prominent pro-angiogenic factor activated by HIF in response to hypoxia is the vascular endothelial growth factor (VEGF-A). Cancer cells secrete VEGF-A as different isoforms that diffuse through the tumour microenvironment and bind to the specific transmembrane receptors VEGFR1, VEGFR-2 and neuropilin-1, mainly located on vascular endothelial cells (ECs) [15,16]. VEGFR-2 is believed to mediate most of the paracrine effects of VEGF-A known to promote angiogenesis: vascular permeability, EC proliferation, migration, survival and association in micro-capillary structures [17]. Moreover, VEGFR-2 has been recently implicated in VEGF-A autocrine loops in EC, that seem to be essential to their survival and to the maintenance of the differentiated state of blood vessels [18].

Induction of VEGF-A expression also occurs under different cellular stresses through HIF-1- independent pathways [19-22]. As an example, VEGF-A expression can be activated by the inositol requiring enzyme 1 (IRE1)-dependent pathway, a branch of the unfolded protein response (UPR), in response to hypoxia or glucose deprivation [21]. Reductive agents such as dithiothreitol (DTT) and homocysteine are also potent inducers of the UPR and of an activating transcription factor 4 (ATF4)-mediated transcription of the VEGF-A gene [23-25]. Besides their effects on VEGF-A expression, thiolic-reducing agents may impair VEGF-A signalling on EC via their free radical scavenging properties. Indeed, these compounds may interfere with the production of reactive oxygen species (ROS) which is necessary to the phosphorylation of extracellular signal-regulated kinase (ERK) downstream of VEGFR-2, and to the mitotic response of EC [26,27]. These effects may significantly impact tumour growth and metastasis [28-31].

Despite some reports on HIF expression, the effects of amifostine on angiogenesis have been poorly studied and the results found in the literature are often controversial. Using the developmental angiogenesis model of the chorio-allantoic membrane (CAM), both pro- and anti-angiogenic effects have been reported [32,33]. The anti-angiogenic effect was attributed to the ability of amifostine to modify the redox status of EC *in vitro*. However, a proliferating effect of the drug was concomitantly observed [34]. Moreover, Grdina *et al*. described an anti-metastatic effect of amifostine in mice developing Sa-NH sarcoma, in association with high serum levels of the angiogenesis inhibitor angiostatin. This observation suggests that amifostine could have anti-angiogenic properties [35,36]. Little is written about the effects of amifostine on VEGF-A expression in these studies and nothing in relation to VEGF-A expression in tumour cells.

In order to better characterize the potential effects of amifostine on tumour angiogenesis, we undertook a systematic study to analyse its effect on VEGF-A expression, production and activity on human cancer and EC.

## Methods

### Reagents

We used culture media obtained from Invitrogen (CA, USA) and Cambrex (MD, USA). Fetal bovine serum (FBS) was obtained from Biowest (Nuaillé, France) and was heat-inactivated before use. Growth Factor Reduced Matrigel was obtained from Becton Dickinson Biosciences (NJ, USA). Bovine serum albumine (BSA), cell-culture grade gelatin, fibronectin, DTT, protamine sulphate, aminoguanidine and amifostine (WR-2721) were purchased from the Sigma Chemical Co (MO, USA). The metabolically active form of amifostine (WR-1065) was synthesized by Dr M. Zanda (CNR, Milan, Italy). The VEGF-A neutralizing polyclonal antibody was obtained from R&D Systems (Minneapolis, USA). Primary antibodies against human HIF-2α and β-actin were obtained from Santa Cruz Biotechnology (CA, USA); anti-human Ku80 from Serotec (Oxford, UK); anti- human HIF-1α from BD Transduction Laboratories (Le Pont de Claix, France); anti-VEGFR-2 from ReliaTech (Braunschweig, Germany); anti- phospho-eIF2α (Ser51), anti-eIF2α and antibodies to p42/44 and their phosphorylated counterparts from Cell Signaling Technology (MA, USA). Primers for ATF4 were as described by Namba *et al*. [37]. Primers for GADD34, CHOP, EDEM and BIP were as previously described [21]. Other primer used for mRNA expression analysis, were reported in Additional File 1 (Table S1). All primers were obtained from Proligo (Paris, France).

### Cell culture

Human mammary carcinoma cells MCF7 and colon carcinoma HCT116 cells were a gift from Dr P Hainaut (International Agency for Research on Cancer, Lyon, France). MCF7 cells and U87 human glioma cells (ATCC, HTB-14) were grown in Dulbecco's modified eagle medium (DMEM) supplemented with 10% FBS. HCT116 cells were grown in McCoy medium, 10% FBS. Human umbilical EC (HUVEC; Clonetics, CA, USA) were propagated up to eight passages on a 0.2% gelatine matrix in endothelial growth medium (EGM-2; Bulletkit, Walkersville, USA). Treatments of tumour cells with amifostine were performed in the presence of 4 mM aminoguanidine (AG), as previously described [10]. AG prevents the catabolization of amifostine by FBS Cu-dependant amine-oxydases into cytotoxic compounds [38]. Amifostine treatments on HUVEC were performed without AG. Hypoxic conditions were obtained at 3% O_2 _in a Heraeus incubator BB-6060.

### Lentiviral vector constructs and MCF7 transduction

MCF7 were transduced using lentivectors expressing the fluorescent marker E-GFP and containing short hairpin RNA sequences against HIF-1α [HIF-1α.small hairpin (sh)RNA] or red fluorescent protein (RFP.shRNA) as described [39]. For transduction, MCF7 cells (5.10^4 ^cells per well in a 24-multiwell plate) were incubated for 24 h in complete medium. Cells were then incubated with viral supernatants from 293T cells for 24 h at 37°C in the presence of protamine sulphate. Transduced cells were sorted out 5 days post-transduction by cytofluorimetry. Enhanced green fluorescent protein (E-GFP) positive cells were used for the experiments.

### ATF4 RNA interference and transient transfection of MCF7

Small interfering RNAs (siRNAs) were purchased from Eurogentec (Liège, Belgium). The sequence of the ATF4-targeting SiRNA (SiATF4) was as previously described [40]. A SiATF4 mutated in three nucleotides served as control (SiMUT, 51). Cells at a 50% density were transfected with 250 nM of SiRNA in OptiMEM using Lipofectamine Plus (Invitrogen, CA, USA). After 24 h, cells were either treated or not treated with amifostine; cells and supernatants were then collected for RNA isolation and protein analysis.

### VEGF-A enzyme-linked immunoadsorbent assay (ELISA)

Cells at 60% confluence were grown in 5-cm diameter dishes for the indicated period of time. VEGF-A concentration was measured in cell-conditioned media using a commercial VEGF-A ELISA kit (R&D Systems, Minneapolis, USA). Assays were performed in triplicate and calibration curves were obtained using human recombinant VEGF-A. The results were analysed using the Softmax Pro4.0 software (Molecular Devices Corporation, CA, USA). Cells were counted using a cell counter (Coulter, Becton Dickinson, NJ, USA).

### Wst-1 metabolic assay

The paracrine effect of amifostine was assayed as follows. MCF7 cells were first incubated for 48 h, either in the presence of AG alone or with both AG- and amifostine. Conditioned media (CM) in these conditions were dialysed at 4°C in order to remove the two chemicals. HUVEC grown in 96-multiwell plates to 80% confluence were then starved for 4 h in DMEM without fetal calf serum (FCS) and incubated for 16 h in the presence of CM. Wst-1 assays (Roche Applied Science, IN, USA) were then performed as follow: Wst-1 reagent was added to the cell medium after a 12 h incubation with CM and absorbance was read 4 h later at 440 nm using a spectrophotometer (Molecular Devices Corporation). Results were analysed using the Softmax Pro 4.0 software (Molecular Devices Corporation). The assay was performed in triplicates. In similar experiments, CM were pre-incubated for 45 min at 37°C with a VEGF-A neutralizing antibody or with an irrelevant anti-ß-actin antibody, prior to addition to HUVEC.

### Polymerase chain reaction analysis (PCR)

Reverse transcription (RT) were performed as previously described [39]. A semi-quantitative analysis of VEGF-A was performed by co-amplifying VEGF-A and β-actin in a Thermal Cycler (Eppendorf AG, Hamburg, Germany) at 94°C for 40 s, 59°C for 30 s, 72°C for 50 s, throughout 35 cycles, with a final elongation step of 3 min at 72°C. Real time quantitative PCR (Q-PCR) analyses were performed using the MX3000p thermocycler (Stratagene, CA, USA) and the SYBRgreen dye (ABgene, Epsom, UK) methodology. The relative abundance of transcripts was calculated by using α-tubulin or β-actin as standards.

### Immunoblot analysis

MCF7 cells and HUVEC were grown up to the subconfluence in 10 cm diameter dishes. HUVEC were starved for 24 h in EBM-2 medium, 2% FCS prior to treatments. Treatments were then performed in fresh medium (EBM-2 for HUVEC, complete culture medium for MCF7) and total protein extracts were collected on ice at different time points with an electrophoretic mobility shift assay buffer-B [11] for HIF and mitogen activated protein kinase analysis or as described by Drogat *et al*. for eIF-2α analysis [22]. Forty to fifty micrograms of proteins were separated in SDS-PAGE gels and transferred to a 0.4 μm nitrocellulose membrane (Schleicher & Schuell, Dassel, Germany). Proteins of interest were detected using specific primary antibodies (see figure legends) and secondary antibodies were coupled to horseradish peroxidase (DAKO SA, Glostrup, Denmark). Blots were revealed using the enhanced chemiluminescence +reagent (Amersham Pharma Biotech, NJ, USA) followed by radioautography or direct quantification of chemiluminescence (Fujifilm LAS-3000).

### VEGF-A radioligand binding studies

VEGF-A_165 _was radio-labelled as previously described [41] to a specific activity ranging from 20,000 to 100,000 cpm/ng. HUVEC were seeded at 7000 cells/cm2 in six-well plates, cultured for 2 days in EGM-2 and starved for 24 h in 2% FBS-containing EBM-2 prior to a 12 h-incubation in the presence, or not, of 1 mM WR-1065. The plates were washed once with phosphate buffered saline (PBS) and the cells were incubated for 2 h at 4°C with 125I-VEGF-A in DMEM, 20 mM HEPES, 0.2% gelatine. They were rinsed three times in PBS and finally solubilized at room temperature in a solution containing 2% Triton X-100, 10% glycerol and 1 mg/mL BSA. Total cell lysates were counted for radioactivity using a Kontron MR 250 gamma-counter. Each value reported in the graph was the mean of duplicate determinations. Unspecific binding was defined as the amount of radioactivity bound in the presence of 20 μg/mL protamine sulphate, which gave results similar to the use of a 100-fold excess of VEGF-A. The experiments were repeated three times with similar results. Binding data were analysed using the GraphPad Prism 4.0 software.

### Transwell cell migration assays

HUVEC cell migration was measured using 24-well-Transwell plates (Becton Dickinson Labware, Le Pont de Claix, France). Prior to cell seeding, inserts (8-μm pore size) were precoated overnight at 4°C on the lower side using 10 μg/mL fibronectin in PBS. Membranes were then blocked 1 h at room temperature with 1% heat-inactivated BSA. HUVEC (10^5 ^cells per well) were seeded in the upper compartment and allowed to migrate for 7 h throughout the membranes in DMEM containing 0.5% FBS and 0.1% BSA and in the presence of different concentrations of WR-1065. VEGF-A (10 ng/mL) was added in the lower compartment as chemo-attractant. After migration, cells that had migrated were fixed and stained in a solution containing 30% methanol, 10% acetic acid and 0.1% Coomassie Blue. The extent of cell migration was analysed by counting cells in at least six random fields per Transwell filter using the LUCIA image analysis software (Laboratory Imaging, Praha, Czech Republic). Results are from three independent experiments.

### Matrigel tube formation assays

Amifostine was tested for its ability to modulate endothelial cell differentiation into tube-like structures using a Matrigel surface assay. Cells (2 × 10^5 ^cells/mL of Growth Factor Reduced Matrigel, BD Biosciences, MA, USA) were seeded in EBM-2 medium supplemented with 0.2% FBS and 1% L-glutamine and allowed to adhere for 1 h. Media were then replaced by EBM-2 enriched with 0.5% FBS, containing increasing concentrations of WR-1065 with or without 10 mg/mL of VEGF-A. Cells were then incubated for 3 h at 37°C. Total length of tube-like structures was assessed using the LUCIA image analysis software. Results shown are representative of three independent experiments. Each data point is the mean of six randomly chosen fields.

### Statistics

Statistical analysis of data was performed using the paired Student's *t *test *P *value (*P < 0.05; **P < 0.01).

## Results

### Amifostine treatment induces VEGF-A expression in cancer cell lines

The potential effects of the cytoprotective drug amifostine on the vascular bed still remains unclear and controversial. In order to better characterize the angiogenesis-related effects of this molecule, we analysed the effects of its dephosphorylated active form (WR-1065) on the mRNA expression of VEGF-A, a growth factor that plays a major role in tumour angiogenesis.

**Figure 1:**
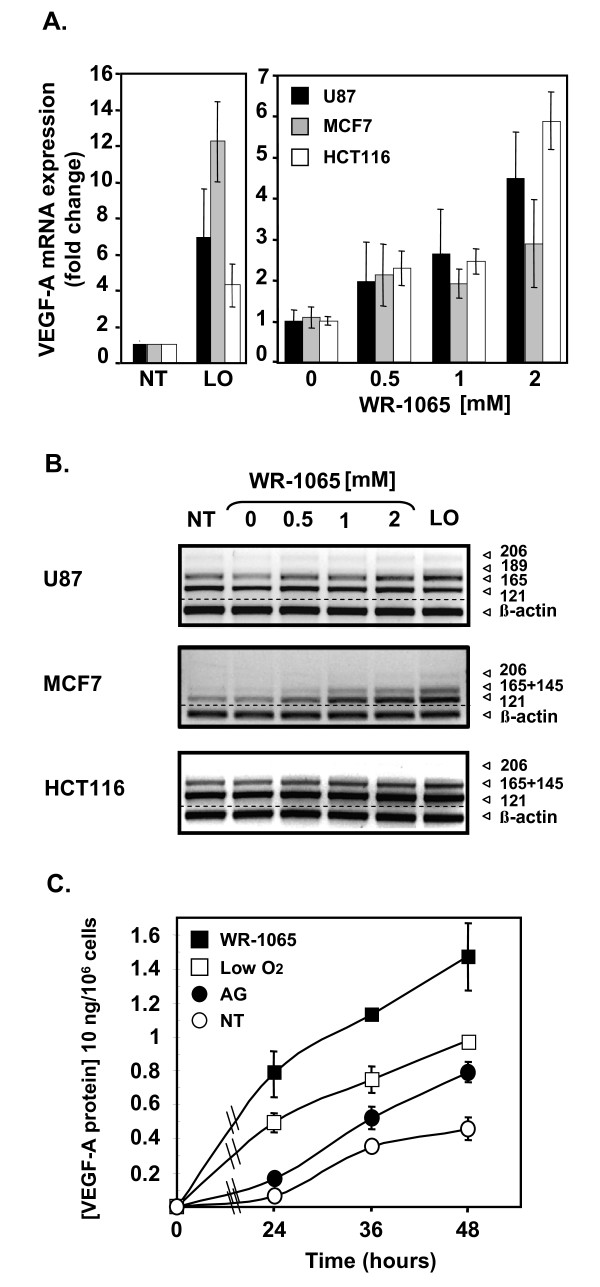
**Amifostine enhances vascular endothelial growth factor A (VEGF-A) mRNA and protein expression in cancer cells**. MCF7 and HCT116 carcinoma cells, as well as U87 glioma cells, were grown up to 70% confluence in 10-cm culture dishes. Cells were then incubated in freshly added complete medium under classical conditions of oxygen (NT, 20% 0_2_) or low oxygen conditions (LO, low O_2_, 3% 0_2_), or in the presence of the indicated concentrations of WR-1065 under 20% of oxygen. (A) Amifostine increases VEGF-A mRNA levels in U87, MCF7 and HCT116 cells. Following a 16-h (MCF7 cells, grey bars) or a 24-h (HCT116 cells, white bars; U87 cells, black bars) incubation time, total mRNA was isolated. Expression of VEGF-A mRNA in cells was measured by quantitative polymerase chain reaction using primers amplifying all the VEGF-A isoforms. Histogram values represent the level of expression of all VEGF-A splice variants, normalized to α-tubulin. Results are the mean values ± standard error of mean of three independent experiments. (B) Amifostine increases specific VEGF-A mRNA isoforms. Shown is a representative gel electrophoresis pattern of the different VEGF-A splice variants in U87, MCF7 and HCT116 cells, β-actin being used as standard. (C) Amifostine increases VEGF-A protein secretion by MCF7 cells. Cells were treated up to 2 days in complete medium, and conditioned media were collected after 24 h, 36 h and 48 h of treatment. VEGF-A protein secretion was measured by ELISA using supernatants of cells grown under low levels of oxygen (3% O_2_,) or 20% of oxygen, and of cells treated with 1 mM WR-1065 in the presence of aminoguanidine (AG) or with AG alone. Results shown are representative of three independent experiments done in triplicates.

Three human tumour cell lines from different tissue origins (U87 glioma cells, MCF7 breast carcinoma cells and HCT116 colon carcinoma cells) were treated with increasing amounts of WR-1065, surrounding 1 mM, which is a dose corresponding to the measured concentration of the drug in tissues of animal models [8,42]. Total VEGF-A mRNA level was determined by quantitative PCR (Q-PCR) and its different isoforms were distinguished by semi-QPCR. As shown in Figure 1A, WR-1065 treatment induces the accumulation of VEGF-A mRNA in a dose-dependent manner in all cell types. This effect was mostly observed for transcripts that correspond to the two diffusible isoforms (VEGF_121, 165_) of the growth factor (Figure 1B). As determined for MCF7 cells, VEGF-A mRNA increase is accompanied by a significant release of VEGF-A protein in the culture medium (Figure 1C). These results suggest a potential pro-angiogenic effect of amifostine.

### VEGF-A mRNA accumulation in response to WR-1065 is independent of HIF

**Figure 2:**
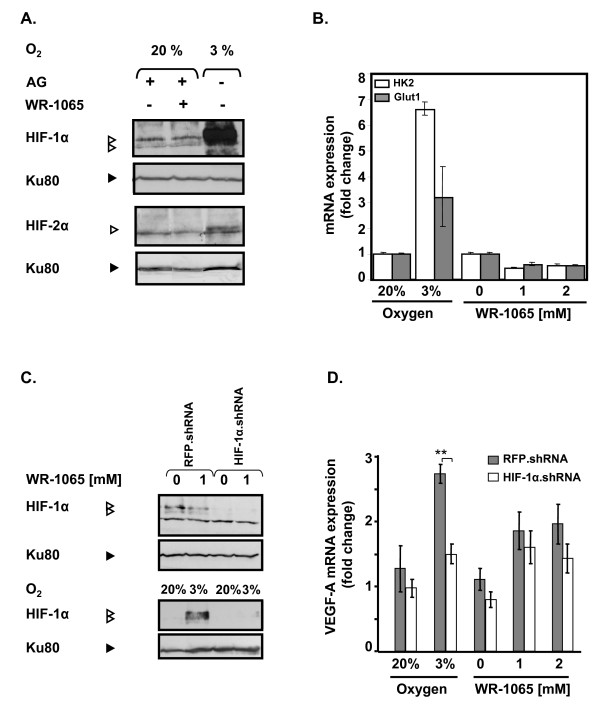
**Amifostine upregulates vascular endothelial growth factor A (VEGF-A) mRNA production independently of hypoxia-inducible factor (HIF)**. MCF7 cells were incubated in complete medium with WR-1065 in the presence of aminoguanidine (AG) or were subjected to low levels of oxygen (3% 0_2_,). (A) Amifostine treatment does not lead to any accumulation of HIF proteins. Cells were treated for 6 h under 20% of oxygen (20% O_2_) with or without 1 mM WR-1065; or low oxygen conditions (3% O_2_). HIF-1α and HIF-2α were detected by Western blot (Ku80 used as internal control). (B) Amifostine treatment does not lead to the induction of HIF-1 target genes. Cells were treated with WR-1065, in the presence of AG, for 16 h or exposed to low oxygen conditions (3% O_2_) for 16 h. Total mRNA were isolated and the expression of *HK-2 *and *GLUT-1 *genes were assessed by quantitative polymerase chain reaction Q-PCR); β-actin served as internal control. (C/D) Amifostine-mediated VEGF-A upregulation is not impaired by a HIF-1α.shRNA (small hairpin RNA). (C) Western blot analysis of HIF-1α protein levels in MCF7 cells transduced with RFP.shRNA (control) and HIF-1α.shRNA constructions. Cells were treated with 1 mM WR-1065 for 16 h (upper panel, "+"), or exposed to low oxygen conditions (3% O_2_) or classical conditions (20% O_2_) for 6 h (lower panel). Proteins were analysed by Western blot using specific antibodies for HIF-1α and for Ku80 (control of loading). (D) VEGF-A mRNA expression was assessed by Q-PCR in MCF7 cells transduced with lentiviral constructs expressing either HIF-1α.shRNA or control RFP.shRNA (grey and white bars, respectively). Cells were incubated for 16 h in the presence of WR-1065 or subjected to low oxygen conditions (3% O_2_). Expression of global VEGF-A mRNA in HIF-1α.shRNA-expressing cells was determined by Q-PCR and expressed in fold-change relative to untransduced-cells left untreated. Significant expression changes were determined by Student's paired tests.

HIF-1α and HIF-2α are involved in the transactivation of VEGF-A gene in response to hypoxia [43] and amifostine has been described as a potent hypoxia-mimetic compound that could activate HIF-1α both *in vitro *and *in vivo *[13]. Therefore, we analysed by Western Blot the accumulation of the hypoxia responsive transcription factors HIF-1α and HIF-2α in MCF7 cells treated with 1 mM WR-1065. As shown in Figure 2A, WR-1065 treatment did not lead to the accumulation of either HIF-1α or HIF-2α in treated cells, whereas a strong increase in HIF-1α was observed in cells subjected to low oxygen concentration (3% instead of 20%). Consistently, 1 or 2 mM WR-1065 failed to induce any nuclear translocation of HIF-1α whereas lowering oxygen led to a significant increase of HIF-1α in the nucleus (Additional File 2, Figure S1).

We then measured the mRNA level of two representative HIF-1α-dependent genes, namely *HK2*, *GLUT1*. As expected, expression of these genes was strongly increased under low oxygen conditions (3% O_2 _Figure 2B). In comparison, their expression was significantly repressed (≈ 50%) in response to WR-1065 treatment, which suggest an HIF-1α-independent effect of WR-1065 on VEGF-A. In order to definitively exclude an HIF-1-dependent activation of VEGF-A by amifostine, we abolished HIF-1α protein expression by stably expressing an shRNA directed against the mRNA of HIF-1α [39]. MCF7 cells were then treated with WR-1065 or subjected to low oxygen conditions (3%). Under low level of oxygen (3%), shRNA.HIF but not shRNA.RFP (Red Fluorescent Protein), led to a strong inhibition of both HIF-1α protein (90%, Figure 2C) and VEGF-A mRNA accumulation (50%, Figure 2D). In response to WR1065 treatment, knock down of HIF-1α did not significantly modify the increase of VEGF-A mRNA expression. Altogether, these results indicate that the accumulation of VEGF-A mRNA in WR-1065-treated cells does not depend on HIF-1α activation.

### Amifostine activates the eIF2α/ATF4 triggering pathway in cancer cells

**Figure 3:**
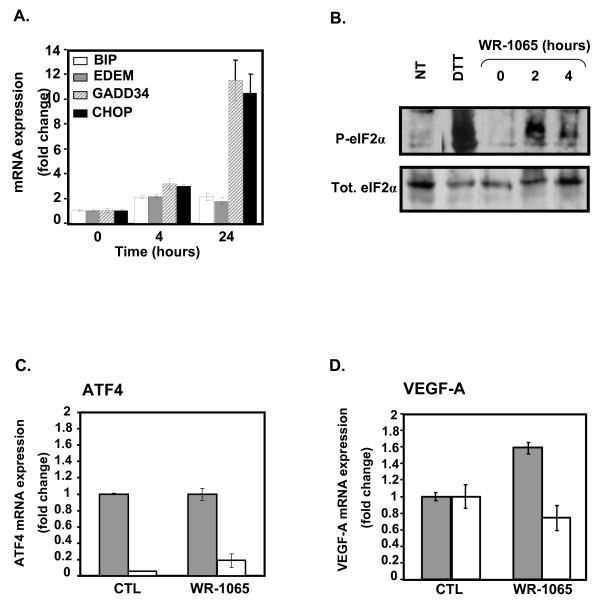
**Amifostine activates the unfolded protein response-dependent signalling pathways**. MCF7 and U87 cells were treated for increasing periods of time with 2 mM WR-1065 or 10 μg/mL Tunicamycin (Tun) in complete culture medium. Proteins and mRNA were collected at several time points, and protein and gene expression were assessed by Western-Blot and polymerase chain reaction (PCR) analyses. (A) Amifostine differentially up-regulates *BIP*, *EDEM, GADD34 and CHOP *genes in MCF7 cells. Quantitative-PCR profiles of *BIP*, *EDEM*, *GADD34 *and *CHOP *genes, in MCF7 cells treated with WR-1065. Results were normalized using α-tubulin mRNA and expressed as ratios between the treated [aminoguanidine (AG)+WR1065] and untreated (AG alone) conditions. The time zero of the experiment is set to 1. (B) Amifostine triggers eIF2α phosphorylation in MCF7 cells. Western-blot analysis of total (Tot.eIF2α) and phosphorylated eIF2α (P-eIF2α) in MCF7 cells treated or not with 2 mM WR-1065. A 1 h treatment with 2 mM dithiothreitol was used as a positive control for eIF2α phosphorylation. Total protein extracts were resolved on 12% SDS-PAGE, and protein levels were assessed using specific anti-eIF2α and anti-P-eIF2α antibodies. (C/D) Amifostine-induced vascular endothelial growth factor A (VEGF-A) up-regulation requires activating transcription factor 4 (ATF4). MCF7 cells were transduced with a small interfering (si)RNA directed against ATF4 (SiATF4, white bars) or with the same siRNA mutated in three nucleotides (SiMUT, grey bars), used as control;. Transduced cells were treated for 24 h with AG alone (CTL) or in combination with 2 mM WR-1065. (C) ATF4 gene expression was assessed by real-time reverse polymerase chain reaction (RT-PCR). (D) VEGF-A gene expression was assessed by real-time RT-PCR. ATF4 and VEGF-A mRNA expression levels in amifostine-treated cells are expressed as fold changes to the control condition (AG alone), which were set to 1 for SiATF4 and SiMUT. Error bars correspond to standard deviations for each triplicate determination.

VEGF-A mRNA induction through HIF-1-independent signalling pathways has already been described in response to a variety of cellular stresses, including the cell response to the accumulation of mis/unfolded proteins (UPR). Since WR-1065 is a thiol reducing agent, we questioned its effect on the UPR triggering pathways. We first analysed the expression of the UPR-target genes *BiP*, *EDEM*, *GADD34 *and *CHOP *in cells treated with WR-1065. As shown in Figure 3A, the expression of the IRE1-dependent genes *EDEM *and *BIP *were slightly (twofold) increased under these conditions. Consistently, the splicing of the XBP1 transcript, another hallmark of IRE1 activation, was neither observed in MCF7 nor in U87 cells treated with WR-1065 (Additional File 3, Figure S2). This confirms that IRE1 was not significantly activated by amifostine. Besides, *GADD34 *and *CHOP *genes were highly up-regulated (10- to 13-fold) in response to WR-1065 treatment, which evokes an activation of the eIF2α/ATF4 dependent pathway. Indeed, eIF2α was transiently phosphorylated after 2 h of treatment with WR-1065 (Figure 3B). Consistently, blockade of ATF4 expression using a siRNA strategy (Figure 3C) led to the inhibition of WR-1065-induced VEGF-A expression, both at the mRNA (80 to 90%, Figure 3D) and at the protein (50%, Additional File 3, Figure S3) levels. Overall, these results indicate that ATF4 is required for amifostine-induced VEGF-A up-regulation.

### Amifostine-treated tumour cells mediated a paracrine stimulation of EC

**Figure 4:**
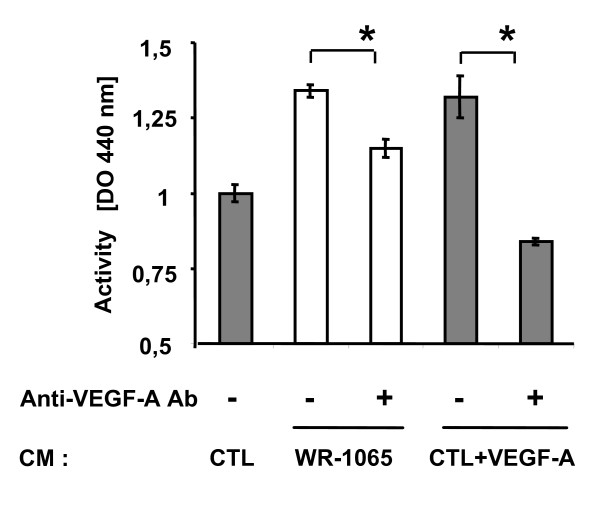
**Amifostine induces a paracrine stimulatory effect on endothelial cells (ECs)**. MCF7 cells were grown to subconfluence and incubated for 48 h in the presence or absence of 1 mM WR-1065. Conditioned media (CM) were then collected and dialyzed to remove WR-1065. Cells were counted. Vascular endothelial growth factor A (VEGF-A) neutralizing antibody (1 μg/mL) were added or not to CM from WR-1065-treated (WR-1065) or AG-treated control cells (CTL). These control cells were supplemented (CTL+VEGF) or not (CTL) with 10 ng/mL VEGF-A. CM were added to HUVEC that have previously been starved in fetal bovine serum-free Dulbecco's modified eagle medium (see Materials and Methods section). Human umbilcal vein EC metabolic activity was measured using the Wst1 assay over a 14 h incubation time in the presence of CM. Results are expressed as means ± standard deviation of the OD (Optical Density) measured at 440 nm from six independent cell culture wells. Significant changes in OD, as determined by Student paired tests, are depicted on the histogram (* *P *< 0.05; ** *P *< 0.01).

The increased release of VEGF-A by WR-1065-treated tumour cells suggests a possible paracrine activity mediated by the chemoprotectant amifostine on EC. In order to confirm this, MCF7 cells were stimulated by WR-1065 for 48 h and cell-conditioned media were then tested for their ability to stimulate starved EC using the MTT metabolic assay (Figure 4). WR-1065 indeed stimulated the release by tumour cells of a diffusible EC growth factor that is able to significantly increase the metabolic activity of EC cells (34%). This stimulatory effect was partly inhibited (20%) by 1 μg of a VEGF-A neutralizing antibody, indicating that VEGF-A produced by tumour cells plays a significant role in this endothelial cell stimulation. Increasing the concentrations of the neutralizing VEGF-A antibody (from 0.03 to 1 μg) failed to produce a higher inhibitory effect, whereas it completely abolished the effect of recombinant VEGF-A. These results suggest that additional EC growth factors may also contribute to the tumour-mediated EC stimulation.

### Inhibitory effects of amifostine on EC

**Figure 5:**
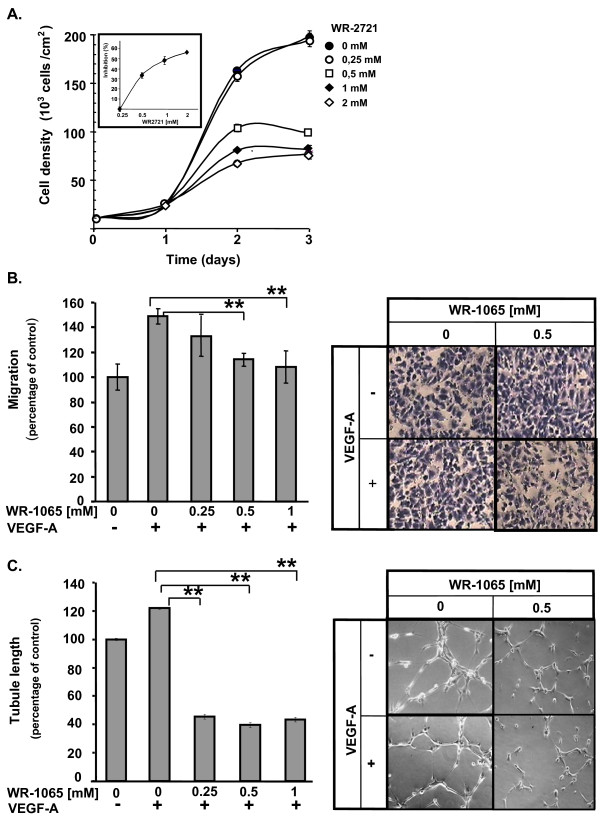
**Amifostine inhibits human umbilcal vein endothelial cells (HUVEC) proliferation and vascular endothelial growth factor A (VEGF-A)-induced migration and differentiation**. (A) Amifostine inhibits HUVEC proliferation. HUVEC growing in endothelial growth medium-2 supplemented with 0, 0.25, 0.5, 1 or 2 mM of WR-2721 were counted for 3 days. The insert shows the inhibition of cell proliferation after 2 days of treatment, in percentage of untreated cells. (B) Amifostine inhibits VEGF-A dependant HUVEC migration. A Transwell migration assay was used. HUVEC were seeded in upper compartments and incubated in Dulbecco's modified eagle medium containing (DMEM) 0.5% fetal bovine serum (FBS), 0.1% bovine serum albumine and increasing concentrations of WR-1065. 10 ng/mL VEGF-A were, or were not, added in the lower compartment and cells were allowed to migrate for 7 h. Cells that migrated were fixed, stained and quantified. Left panel: quantification of VEGF-A-dependent HUVEC migration (% of the migration versus control untreated cells). Significant changes were determined by Student paired tests (*P < 0.05; **P < 0.01). Right panel: corresponding photomicrographs of HUVEC. (C) Amifostine inhibits VEGF-A induced capillary-like structures formation. HUVEC were seeded on GFR-Matrigel. After a 1 h incubation in 0.5% FBS-containing DMEM to allow cell adhesion, cells were treated with WR-1065 for 3 h, in the presence or absence of 10 ng/mL VEGF-A. Total tubule length was determined using NIS image analysis software. Left panel: quantification of total tubule length in the different experimental conditions. Results are expressed as percentages of the average tubule length in the control condition, set to 100%. The control condition corresponds to absence of both VEGF-A and WR-1065. Significant changes, were determined by Student paired tests (*P < 0.05; **P < 0.01). Right panel: phase-contrast microphotographs of tubule networks formed by HUVEC, at a 200-fold magnification.

The stimulatory paracrine effect of VEGF-A on EC should predict stimulation of the neovascularization process. However, since EC cells are the first cellular target of amifostine *in vivo*, we also considered the effect of amifostine directly on EC proliferation, migration and differentiation into tubular-like structures. Thus, HUVEC were grown in the presence of VEGF-A with various concentrations of WR-2721. This allows a progressive release of WR-1065 throughout the duration of treatment. Compared to untreated cells, WR-2721-treated cells have a significantly reduced cell growth and the effect was dose-dependent (Figure 5A, ED_50 _~ 0.5 mM after a 2-day incubation, see insert). This growth-inhibition was also observed using bovine EC of aortic origin (BAE; Additional File 4, Figure S4A). The inhibitory activity may result from a p53-dependent cell cycle arrest in G_1 _as previously described ([10], see also Additional File 4, Figure S5). We then determined whether amifostine may influence VEGF-A-induced EC migration. HUVEC were treated, or not, with VEGF-A in the presence of increasing concentrations of WR-1065 and allowed to migrate for 7 h. Figure 5B shows that WR-1065 inhibits the VEGF-A-induced migration of EC in a dose dependent manner. Again, similar result was obtained using BAE cells (Additional File 4, Figure S4B). Finally, we analysed the effects of amifostine treatment on the *in vitro *formation of capillary-like structures by EC. For this, HUVEC were cultured on Matrigel and treated or not with VEGF-A and/or WR-1065. Figure 5C shows that treatment with WR-1065 completely abolished the VEGF-A induced capillary formation. WR-1065 alone had no significant effect, neither on migration, nor on tubule formation on serum-starved EC (data not shown). Taken together these results indicate that amifostine strongly impairs the activity of VEGF-A on EC proliferation, migration and differentiation, making them unresponsive to a potential paracrine effect mediated by this growth factor.

### Amifostine treatment impairs VEGF-A binding and signalling in EC

**Figure 6:**
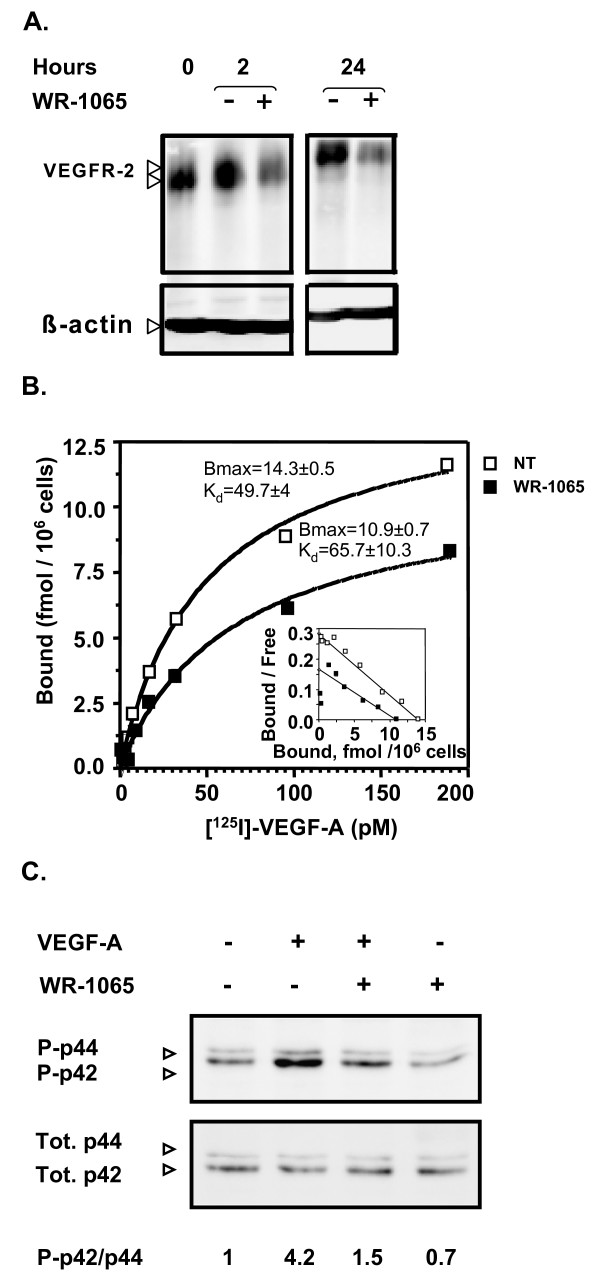
**Amifostine inhibits vascular endothelial growth factor (VEGF)R2 expression, VEGF-A binding and signalling in human umbilcal vein endothelial cells (HUVEC)**. A Amifostine treatment inhibits VEGFR-2 protein expression in HUVEC. After a 24 h-starvation, subconfluent HUVEC were incubated in the presence or absence of 1 mM WR-1065 for indicated period of time and total proteins were collected in an RIPA buffer and resolved on a 7.5% acrylamide SDS-PAGE gel. Proteins were blotted onto a nitrocellulose membrane and probed with a mouse monoclonal antibody against VEGFR-2. Equal loading of proteins was confirmed by β-actin detection. VEGFR-2 migrated at gel positions corresponding to the 150- and 200-kDa forms, as already reported in HUVEC. The blot is representative of three independent experiments. (B) Amifostine treatment impairs VEGF-A binding on HUVEC. HUVEC monolayers (7000 cells/cm2) were allowed to grow for 3 days and then incubated for 12 h in the absence or presence of 1 mM WR-1065. Cells were then exposed to increasing concentrations of 125I-labelled VEGF-A for 2 h at 4°C. The amount of specifically bound iodinated VEGF-A was then determined. Insert represents Scatchard plot corresponding to the saturation curves. The experiment was carried out three times with similar results. (C) Amifostine treatment impairs mitogen activated protein kiinase signalling in HUVEC. Subconfluent HUVEC in serum-free Dulbecco's modified eagle medium were treated with 10 ng/mL VEGF-A for 10 min, alone or together with 1 mM WR-1065. Cytoplasmic proteins were then collected and were resolved on 15% SDS-PAGE. Total (phosphorylated and unphosphorylated) p42/p44 proteins as well as the phosphorylated proteins (P-p42/P-p44) were probed using specific antibodies. Signal was assessed by quantification of chemoluminescence, and ratios between signal intensities of P-p42/P-p44 and total p42/p44 are given beneath the gel. They were normalized to the ratio obtained for untreated cells (VEGF -, WR-1065-), which is set to 1.

In order to understand how amifostine does inhibit EC response to VEGF-A, HUVEC were treated with WR-1065 and the level of VEGF receptor 2 (VEGFR-2) was measured by Western blot. Figure 6A shows that from 2 h to 24 h of incubation with WR-1065, VEGFR-2 protein expression is decreased by ~50% in treated cells as compared to untreated cells. This down-regulation occurs most likely at different levels including the transcriptional level, since VEGFR-2 mRNA is transiently down-regulated in these conditions (Additional File 5, Figure S6). Scatchard analysis of 125I-VEGF-A binding to its receptors on HUVEC was also performed. In agreement with the decrease in VEGFR-2 protein expression, the binding data of three independent experiment consistently showed a ~25% to 30% decrease in the number of VEGF-A molecules associated to high affinity receptors after a 12 h pre-treatment with 1 mM of WR1065 (Figure 6B). The apparent dissociation constant was not significantly modified in these conditions, which indicates that the decrease in VEGF-A binding is due in part to the down regulation of its receptors. In addition to this long-term effect of amifostine on VEGFR2 expression and availability at the cell membrane, immediate phosphorylation of p42/p44 kinase in HUVEC in response to VEGF-A was strongly reduced (~64%) when applied in the presence of WR1065 (Figure 6C). This was also observed to a greater extent in BAE cells (Additional File 4, Figure S4C). Together, these results indicate that WR-1065 has both immediate and long-term effects on the EC responses by impairing VEGF-A binding and signalling.

## Discussion

Our investigations showed that the radio- and chemo-protective drug amifostine is a potent inducer of VEGF-A expression, acting both at the mRNA and protein levels in several human cancer cell lines. Accumulation of VEGF-A mRNA was detected at concentrations of the drug (0.5 - 2 mM) that correspond to those measured in most tissues of treated rats and monkeys 10 to 30 min after an injection of a single protective dose [8,42]. This suggests that VEGF-A up-regulation may also occur along clinical cancer treatments of patients with the cytoprotective drug and questions about the possible angiogenic side effects of such treatments.

To date, the effects of amifostine on angiogenesis were still poorly studied and results were found somewhat controversial [32]. In addition, little is known of the effects of amifostine on VEGF-A expression. Therefore, the potential impact of amifostine on angiogenesis has to be clarified, in particular in relation to the VEGF-A expression. A study from Koukourakis showed that HIF-1α was stabilized both *in vitro *and *in vivo *by high concentrations of amifostine (8 mM) [13]. Since HIF-1α acts as a pro-angiogenic transcription factor and increases VEGF-A expression [14], we first thought that it was responsible for VEGF-A up-regulation in cancer cells exposed to amifostine in our experiments. However, using concentrations of amifostine consistent with the clinical approach, we failed to observe in our cell models any accumulation of either HIF-1α or HIF-2α proteins in response to the drug treatment. Our data clearly show that HIF-1α activity is not involved in VEGF-A up-regulation under amifostine treatment at clinical doses, which led us to consider alternative VEGF-A stimulatory pathways.

Amifostine is a disulphide reducing agent that can generate redox stress in cells [12] and other thiol-containing compounds are known to affect protein folding and to activate the UPR [24]. We therefore investigated whether UPR was activated in response to amifostine and whether this could lead to VEGF-A mRNA expression as previously reported [21]. The increased expression of several genes considered to be hallmarks of the UPR pathways (*BIP, EDEM, CHOP *and *GADD34*) following amifostine treatment suggests that amifostine did activate the UPR signalling pathways. However, the low increase in *BIP *and *EDEM *mRNA expression, which usually relies upon IRE1- and ATF6-dependant pathways [44,45], suggests that these pathways were not fully activated in response to amifostine. In addition, there was no detectable XBP1 splicing in treated cells, suggesting that IRE1 is not induced by amifostine and thus does not significantly contribute to VEGF-A up-regulation.

The strong increase in *CHOP *and *GADD34 *mRNA expression could be linked to either PERK activation or to the GCN2-dependant pathway, both leading to eIF2α phosphorylation, inhibition of cap-dependant translation and activation of the ATF4 transcription factor [46,47]. Interestingly, homocysteine, a compound related to amifostine by the presence of its thiol function, has been shown to induce ATF4 transcriptional activity, leading to increased expression of VEGF-A [24]. Consistently, we observed a transient eIF2α phosphorylation in response to amifostine treatment, which suggests the involvement of ATF4 for VEGF-A expression. Indeed, abolition of ATF4 expression (~95%) using siRNA strategy clearly demonstrated that VEGF-A up-regulation upon exposure to amifostine strongly relies on ATF4. However, other pathways may also contribute to the amifostine-mediated effects on VEGF-A expression such as the c-jun N terminal kinase pathway (JNK). JNK is a known intermediate of VEGF-A mRNA and protein up-regulation [48,49] and we previously showed that this pathway is also activated by amifostine [11]. Consistently, the chemical JNK inhibitor SP-600125 did reduce the extent of amifostine-induced VEGF-A activation by 1.4 fold (not shown). Interestingly, JNK activation is also linked to the UPR downstream of the ER-stress-induced activation of IRE1α, independently of its endoribonuclease activity [50], and IRE1α is itself involved in the up-regulation of VEGF-A in cancer cells undergoing ischemia [21]. Collectively, these data suggest that JNK might also synergize with ATF4 to up-regulate VEGF-A expression in response to amifostine.

The potential pro-angiogenic effect of amifostine in cancer cells may not be limited to the activation of VEGF-A expression. Experiments using VEGF-A blocking antibodies indicated that, in addition to this growth factor, treated tumour cells may also secrete other endothelial cell growth promoting factors. EGF or IL8 are possible candidates for this effect stand, and their expressions were up-regulated (up to 13- and 5.2-fold, respectively) in MCF7 cells upon stimulation by amifostine, as determined using transcriptome analysis (not shown). Amifostine is currently used as a cytoprotector in therapeutic treatment, either as a single dose treatment for cancer therapies or in long-term chronic delivery in the treatment of myelodysplasic syndromes [4,7,51]. Side-induction of VEGF-A, or of any other pro-angiogenic factors, by cancer cells during these treatments may have detrimental effects on the therapeutical purposes. Therefore, it was necessary to investigate whether amifostine could develop secondary effects when applied directly on EC.

EC are subjected to the highest concentrations of amifostine following its intravenous administration. Comparatively, cancer cells accumulate lower concentrations of the drug, as it is known to hardly penetrate tumour masses [4,8]. Since we had previously found an anti-proliferative effect of amifostine on normal and cancer cells ([10,52] and unpublished data), we considered the possibility that amifostine also impedes EC proliferation, which could overcome the tumour-mediated pro-angiogenic effect. In fact, as reported here, each of the endothelial cell responses to VEGF-A was strongly inhibited by amifostine treatment, therefore suggesting that EC were indeed made unresponsive to tumour-mediated VEGF-A stimulation by clinical doses of the drug.

The origin of EC desensitization in response to amifostine treatment was then considered. The EC proliferation arrest should be considered as the consequence of a p53 dependent-cell cycle arrest in G1, as already observed in various cell types ([10,52] and unpublished data). In addition, we showed that amifostine plays a role in EC capacity to respond to VEGF-A. An immediate impairment of intracellular signalling was observed downstream to this interaction, as indicated by the 50% decrease of the VEGF-A-induced phosphorylation of the ERK kinases in the presence of amifostine. As ROS are essential second messengers in EC [26,27], this inhibitory effect may depends on the ROS scavenging properties of the drug, leading to the blockade of the downstream signalling of VEGFR-2.

In addition to this immediate effect, we observed a long-term effect of amifostine on the capacity of EC to bind VEGF-A. This was shown by a significant decrease in VEGF-A binding to its specific cell-surface receptors after 12 h of amifostine treatment. This long-term effect depends on the down-regulation of VEGFR-2 protein expression at the plasma membrane. This down regulation probably results from a combination of complex intra- and extra-cellular redox effects due to the free thiol group of WR-1065. Part of these effects may occur at the transcriptional level by inactivating redox sensitive transcription factors involved in VEGF-R2 expression as it does for p53 and NFKappa B [10,12,53]. An additional effect may occur at the translational level through the global inhibition of translation due to eIF2a phosphorylation in response to treatment [46,47]. In addition, although no modulation of VEGF-A affinity to its receptors was observed in our experiments, we cannot completely rule out the possibility of an additional direct effect of the thiol group of amifostine on VEGF-A binding to its receptors. Indeed, VEGF-A exists as a disulphide-bond functional dimer. VEGF-A dimerization, which is essential for its biological activity, may be impaired under reducing conditions [54]. Moreover, VEGFR-2 ligand binding sites are located within Ig-like extracellular domains that may also be sensitive to redox conditions [17]. By potentially modifying the structure of the two partners, amifostine may inhibit the interaction between VEGF-A and VEGF-R2, therefore inducing a down-regulation of VEGF-R2 expression [55]. Finally, activation of the stress induced kinase JNK by amifostine may trigger specific phosphorylation of VEGF-R2 receptor that could lead to its internalization and degradation as it has already been described for epidermal growth factor receptor [56]. Thus, several characteristics of amifostine likely contribute to render endothelial cell unresponsive to external VEGF-A stimuli.

## Conclusion

This study shows that amifostine exerts a complex dual role on VEGF-A biology. On the one hand, the drug activates VEGF-A production by cancer cells. On the other hand, it blocks the VEGF-A responsiveness of EC. This latter effect is due to both an immediate effect on VEGF-A binding and/or downstream signalling through VEGFR-2; and to a long-term inhibition of the VEGFR-2 expression at the cell membrane. Since amifostine is believed to enter normal cells better than cancer cells *in vivo *[4,8], and since it is often administered intravenously, its effects should be prominent on EC. This suggests that the drug may act primarily as an anti-angiogenic compound on EC, in addition to its well-characterized protective effects. *In vivo *experiments are on the way to determine whether the use of amifostine does modify the tumour vasculature and/or facilitate the penetration of classical anti-cancer drugs into tumour masses [57].

## List of Abbreviations

AG: aminoguanidine; AFT4: activating transcription factor 4; BSA: bovine serum albumine; CAM: chorio-allantoic membrane; CM: conditioned media; DMEM: Dulbecco's modified eagle medium; DTT: dithiothreitol; EC: endothelial cells; ECL: enhanced chemiluminescence; EGFP: enhanced green fluorescent protein; EGM: endothelial growth medium; ERK: extracellular signal-regulated kinase; FBS: fetal bovine serum; FCS: fetal calf serum; HIF: hypoxia-inducible factor; HUVEC: human umbilical vein EC; IRE: inositol requiring enzyme; JNK: c-Jun N-terminal Kinase; MAPK: mitogen activated protein kinase; PBS: phosphate buffer saline; PCR: polymerase chain reaction; Q-PCR: quantitative PCR; ROS: reactive oxygen species; RT: reverse transcription; shRNA: small hairpin RNA; siRNA: small interfering RNA; UPR: unfolded protein; VEGF-A: vascular endothelial growth factor A.

## Competing interests

The authors declare that they have no competing interests.

## Authors' contributions

SD performed most of the technical part of the study, cell culture and treatment, PCR, ELISA and *in vitro *angiogenesis tests. XC performed VEGF-A binding studies on HUVECs. HR and FM performed viral constructs and viral transduction on MCF7 cells to block HIF1-alpha expression. MB performed WB on Eif2alpha protein and helped in the UPR pathway study. RS and MZ synthesized WR1065 necessary for the study. MM and AB provided scientific support and advised on UPR stress and angiogenesis studies. SN is the principal investigator of this study and performed the cell cycle analysis, the ATF4 part of the experiment, a part of the quantitative RT PCR, the writing and revision of the paper.

## Pre-publication history

The pre-publication history for this paper can be accessed here:

http://www.biomedcentral.com/1741-7015/8/19/prepub

## Supplementary Material

Additional file 1 **Table S1**. Sequences of the primer used either for semi quantitative polymerase chain reaction (Q-PCR) or for real time Q-PCR(*). Primers for activating transcription factor 4 were as described by Namba *et al*. [37]. Primers for GADD34, CHOP, EDEM and BIP were as previously described [21]. All primers were obtained from Proligo (Paris, France). Click here for file

Additional file 2 **Figure S1. Amifostine does not induce hypoxia-inducible factor (HIF)-1α nuclear accumulation**. MCF7 grown to 70% confluence on Lab-Tek chamber slides were treated for 6 h with 4 mM aminoguanidine alone (O_2 _20%) or in combination with 1 mM amifostine (WR-1065), or submitted to low oxygen conditions for 6 h (O_2 _3%). Detection of HIF-1α was achieved by incubation with an anti-human HIF-1α monoclonal antibody. Nuclei were stained by Hoechst 33342. Fluorescence labelling was observed by confocal microscopy, right panel shows the two merged pictures obtained. Click here for file

Additional file 3 **(Figure S2) Amifostine does not induce XBP1 mRNA splicing and (Figure S3) activating transcription factor 4 (AFT4) is required for increased vascular endothelial growth factor A (VEGF-A) protein secretion in response to amifostine**. (Figure S2) MCF7 and U87 cells were grown up to 70% confluence in 10 cm-dishes. Cells were then treated for increasing periods of time with 2 mM WR-1065 or 10 μg/mL Tunicamycin (Tun) in complete culture medium. mRNA were collected at several time points, and mRNA splicing and expression were assessed by reverse transcriptase-polymerase chain reaction analysis. Gel electrophoresis patterns of expression of unspliced (XBP1u) and spliced (XBP1s) XBP1 transcripts, in MCF7 cells and U87 cells. (Figure S3) MCF7 cells were transduced with a small interfering (si)RNA directed against ATF4 (SiATF4, white bars) or with the same siRNA mutated in three nucleotides (SiMUT, grey bars), used as control. Transduced cells were treated for 24 h with aminoguanidine alone (CTL) or in combination with 2 mM WR-1065. VEGF-A protein expression was quantified by ELISA from cell supernatants. Error bars correspond to standard deviations for each triplicate determination. See Additional File 2 for materials and methods for supplementary figures. Click here for file

Additional file 4 **Figures S4 and S5**. (Figure S4) Amifostine inhibits the proliferation and migration of BAE cells, as well as VEGF-A signalling in those cells. Figure S5. Amifostine inhibits cell cycle progression of BCE in G1. See additional file 2 for materials and methods for supplementary figures. Click here for file

Additional file 5 **Figure S6**. Amifostine treatment decreases vascular endothelial growth factor-R2 mRNA expression or stability. After a 24 h-starvation, subconfluent human umbilcal vein endothelial cells were incubated in the presence or absence of 1 mM WR1065 and total mRNA were collected at the indicated time points. Reverse transcription and real time quantitative polymerase chain reaction was performed as previously described (Methods). Results are shown in fold induction as compared to control untreated cells. They were normalized to α-tubulin and correspond to the mean values ± standard deviation of triplicates from three independent experiments. See Additional File 2 for materials and methods for supplementary figures. Click here for file
