# Supplementary material for: The cytoprotective drug amifostine modifies both expression and activity of the pro-angiogenic factor VEGF-A
Source: BMC Med. 2010 Mar 24;8:19. doi: 10.1186/1741-7015-8-19 (PMC2859403; doi:10.1186/1741-7015-8-19)
Supplement: Additional file 1 — Table S1. Sequences of the primer used either for semi quantitative polymerase chain reaction (Q-PCR) or for real time Q-PCR(*). Primers for activating transcription factor 4 were as described by Namba et al. [37]. Primers for GADD34, CHOP, EDEM and BIP were as previously described [21]. All primers were obtained from Proligo (Paris, France). [file 1741-7015-8-19-S1.DOC]

Table 1

| Genes | Primers |
| --- | --- |
| VEGF-A | forward 5'-GCAGCTACTGCCATCCAATC-3'  reverse 5'-GGCGAATCCAATTCCAAGAG-3' |
| ß-actin | forward 5'-TCCATCGTCCACCGCAAATG-3'  reverse 5'-CAATGCTATCACCTCCCCTGTGTG-3' |
| ß-actin* | forward 5’-CGTACCACTGGCATCGTGAT-3’  reverse 5’-GTGTTGGCGTACAGGTCTTTG-3’ |
| VEGF-A* | forward 5’-CGAAACCATGAACTTTCTGC-3’  reverse 5’-CCTCAGTGGGCACACACTCC-3’ |
| HIF-1α* | forward 5'-GGAGATGTTAGCTCCCT-3'  reverse 5'-AGTGGTGGCAGTGGTA-3' |
| Alpha-tubulin* | forward 5'-GAGTGCATCTCCATCCACGTT-3'  reverse 5'-TAGAGCTCCCAGCAGGCATT-3' |
| Glut-1* | forward 5’-GCTACAACACTGGAGTCATC-3’  reverse 5’GGATCAGCATCTCAAAGGAC-3’ |
| HK2* | forward 5’-GCTGGCCGATCAACACCGT-3’  reverse 5’-GCCGTCCGGGGTAGCACA-3’ |
| VEGF-R2* | forward 5’-GCAGGGGACAGAGGGACTTG-3’  reverse 5’-GAGGCCATCGCTGCACTCA-3’ |

**Table 1** Supplemented data : sequences of the primer used either for semi quantitative PCR, or for real time quantitative PCR(*).Primers for ATF4 were as described by Namba et al [3]. Primers for GADD34, CHOP, EDEM and BIP were as previously described [2]. All primers were obtained from Proligo (Paris, France).
