# Supplementary material for: The cytoprotective drug amifostine modifies both expression and activity of the pro-angiogenic factor VEGF-A
Source: BMC Med. 2010 Mar 24;8:19. doi: 10.1186/1741-7015-8-19 (PMC2859403; doi:10.1186/1741-7015-8-19)
Supplement: Additional file 2 — Figure S1. Amifostine does not induce hypoxia-inducible factor (HIF)-1α nuclear accumulation. MCF7 grown to 70% confluence on Lab-Tek chamber slides were treated for 6 h with 4 mM aminoguanidine alone (O2 20%) or in combination with 1 mM amifostine (WR-1065), or submitted to low oxygen conditions for 6 h (O2 3%). Detection of HIF-1α was achieved by incubation with an anti-human HIF-1α monoclonal antibody. Nuclei were stained by Hoechst 33342. Fluorescence labelling was observed by confocal microscopy, right panel shows the two merged pictures obtained. [file 1741-7015-8-19-S2.PDF]

Materials and methods for supplementary figures

Cell culture - Bovine capillary EC (BCE) and bovine aortic cells (BAE), both provided by D.B.Rifkin (NYU, USA), were expanded in DMEM, 1 g/l glucose, supplemented with 10 % FBS, Lglutamine, antibiotics and 2 ng/ml b-FGF2. Those cells were used at passages 4 to 9 in all experiments. Treatments of BAE and BCE with amifostine were performed in the presence of 2 mM

amino-guanidine (AG). Experiments in hypoxic conditions were performed at 3% O_2 in a Heraeus incubator BB-6060.

Immunocytochemistry on HIF1α - Treatments were performed on MCF7 seeded at 85000 cells/well on glass 4-well Lab-Tek chamber slides (Nunc, Thermo Fischer Scientific Inc). Cells were incubated in normoxia with 4 mM AG (NT) alone or in combination with Amifostine 1 mM (WR-1065) for 6h. Positive control was achieved by incubating untreated cells in hypoxic conditions (3% O_2). Cells were then fixed and HIF-1α was labelled using a monoclonal antibody for HIF1α (Transduction Laboratories, BD Biosciences) and a fluorescent secondary antibody (Alexa Fluor 647 from Molecular Probes). Nuclei were stained using Hoechst 33342 dye (Molecular Probes) and cells were observed using a confocal microscope (Leica SP5).

Cell cycle analysis – BCE inoculated at 10000 cells/cm^2 were allowed to grow in complete culture medium supplemented with 2 mM AG and several doses of amifostine (WR-2721), for increasing periods of time. At each time point, cells were trypsinized, centrifuged, washed with

PBS, and prepared following the manufacturer's instructions of the Cycle Test Plus staining kit TM (BD). Analysis was performed on FACScalibur, using the Cell Quest software (BD).

Wound healing cell migration assay This assay was performed following the method described by Sato and Rifkin (40). Briefly, cells were allowed to grow until forming a confluent monolayer. After a 24h-serum starvation in DMEM supplemented with 1% L-Glutamine, cells were treated with aminoguanidine (AG) alone or AG and amifostine (WR-2721) in the presence or absence of 25 ng/ml VEGF-A. At the time of treatment, a wound was made using a thin tip, and fields were delimited by marks done under the wells. A first series of photographs of the wound zone was taken on an inverted microscope, using the LUCIA image analysis software. Cells were allowed to migrate for 14 to 16h, fixed with glutaraldehyde and stained with May-Grunwald and Giemsa. A second series of microphotographs was done, and migrated cells were quantified by comparison of the two series of photographs for each field. Cells were counted in at least four randomly chosen fields. Results shown are representative of three independent experiments.

ATF4 RNA interference and VEGF-A ELISA on MCF7 cells

Small interfering RNAs (siRNAs) were purchased from Eurogentec (Liège, Belgium). Sequence of the ATF4-targeting SiRNA (SiATF4) was as previously described [39]. A SiATF4 mutated in three nucleotides served as control (SiMUT, 51). Cells at a 50% density were transfected with 250 nM of SiRNA in OptiMEM using Lipofectamine Plus (Invitrogen). After 24h, cells were treated or not with amifostine ; supernatants were then collected for VEGF-A ELISA using a commercial VEGF-A ELISA kit (R&D Systems, Minneapolis, USA) as described in Material and Methods.

PCR for Xbp1 splicing were done as previously described [20]

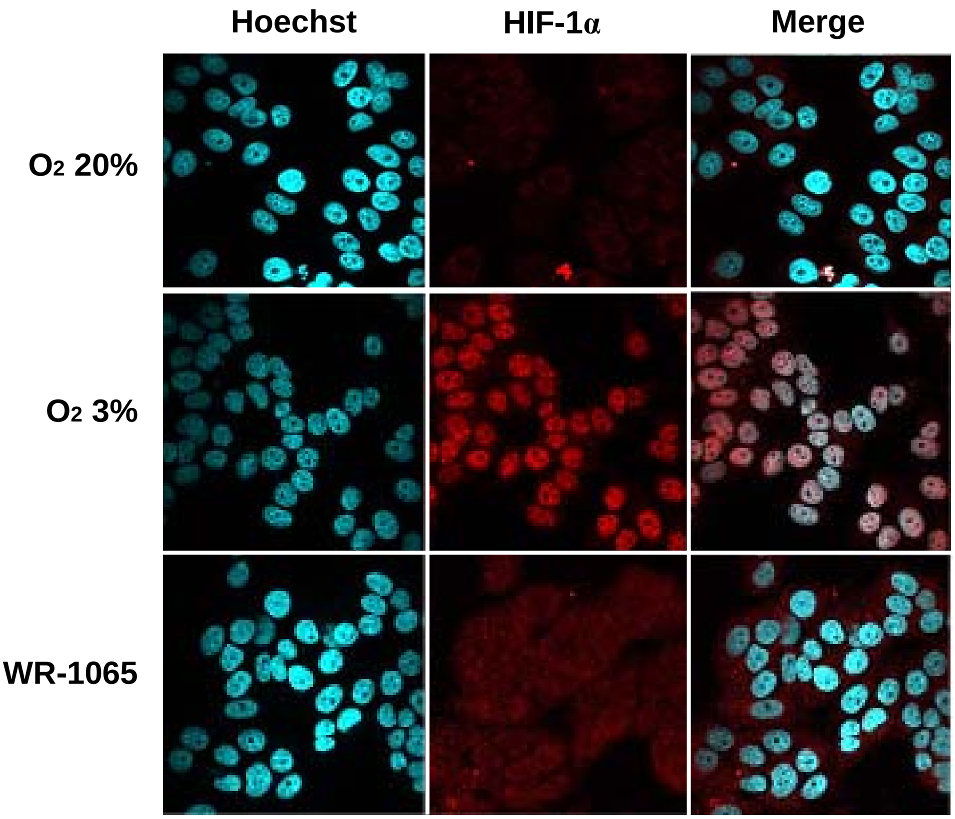

Dedieu et al., Figure S1
