# Supplementary figures and images for: The cytoprotective drug amifostine modifies both expression and activity of the pro-angiogenic factor VEGF-A
Source: BMC Med. 2010 Mar 24;8:19. doi: 10.1186/1741-7015-8-19 (PMC2859403; doi:10.1186/1741-7015-8-19)

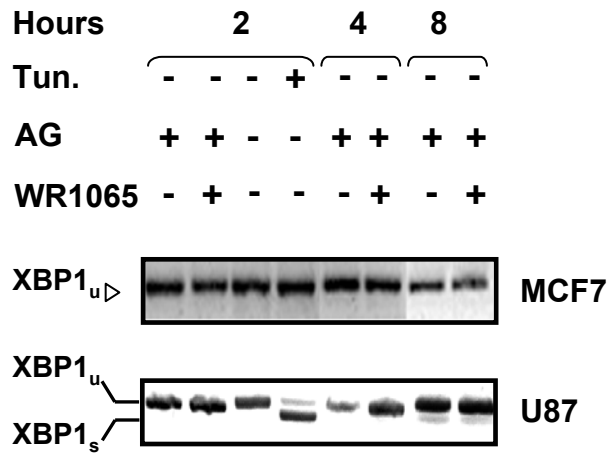

Dedieu et al., Figure S2

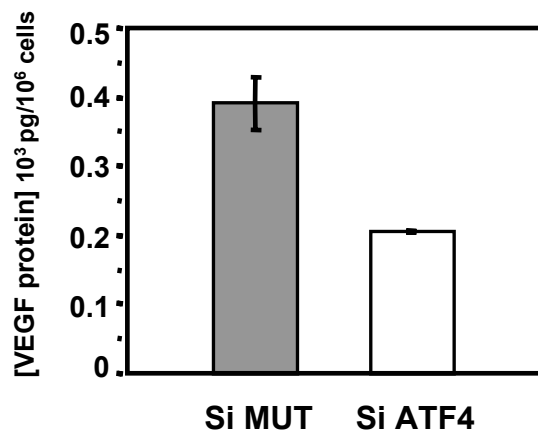

Dedieu et al., Figure S3

Supplement: Additional file 3 — (Figure S2) Amifostine does not induce XBP1 mRNA splicing and (Figure S3) activating transcription factor 4 (AFT4) is required for increased vascular endothelial growth factor A (VEGF-A) protein secretion in response to amifostine. (Figure S2) MCF7 and U87 cells were grown up to 70% confluence in 10 cm-dishes. Cells were then treated for increasing periods of time with 2 mM WR-1065 or 10 μg/mL Tunicamycin (Tun) in complete culture medium. mRNA were collected at several time points, and mRNA splicing and expression were assessed by reverse transcriptase-polymerase chain reaction analysis. Gel electrophoresis patterns of expression of unspliced (XBP1u) and spliced (XBP1s) XBP1 transcripts, in MCF7 cells and U87 cells. (Figure S3) MCF7 cells were transduced with a small interfering (si)RNA directed against ATF4 (SiATF4, white bars) or with the same siRNA mutated in three nucleotides (SiMUT, grey bars), used as control. Transduced cells were treated for 24 h with aminoguanidine alone (CTL) or in combination with 2 mM WR-1065. VEGF-A protein expression was quantified by ELISA from cell supernatants. Error bars correspond to standard deviations for each triplicate determination. See Additional File 2 for materials and methods for supplementary figures. [file 1741-7015-8-19-S3.PDF]

## Slide 1
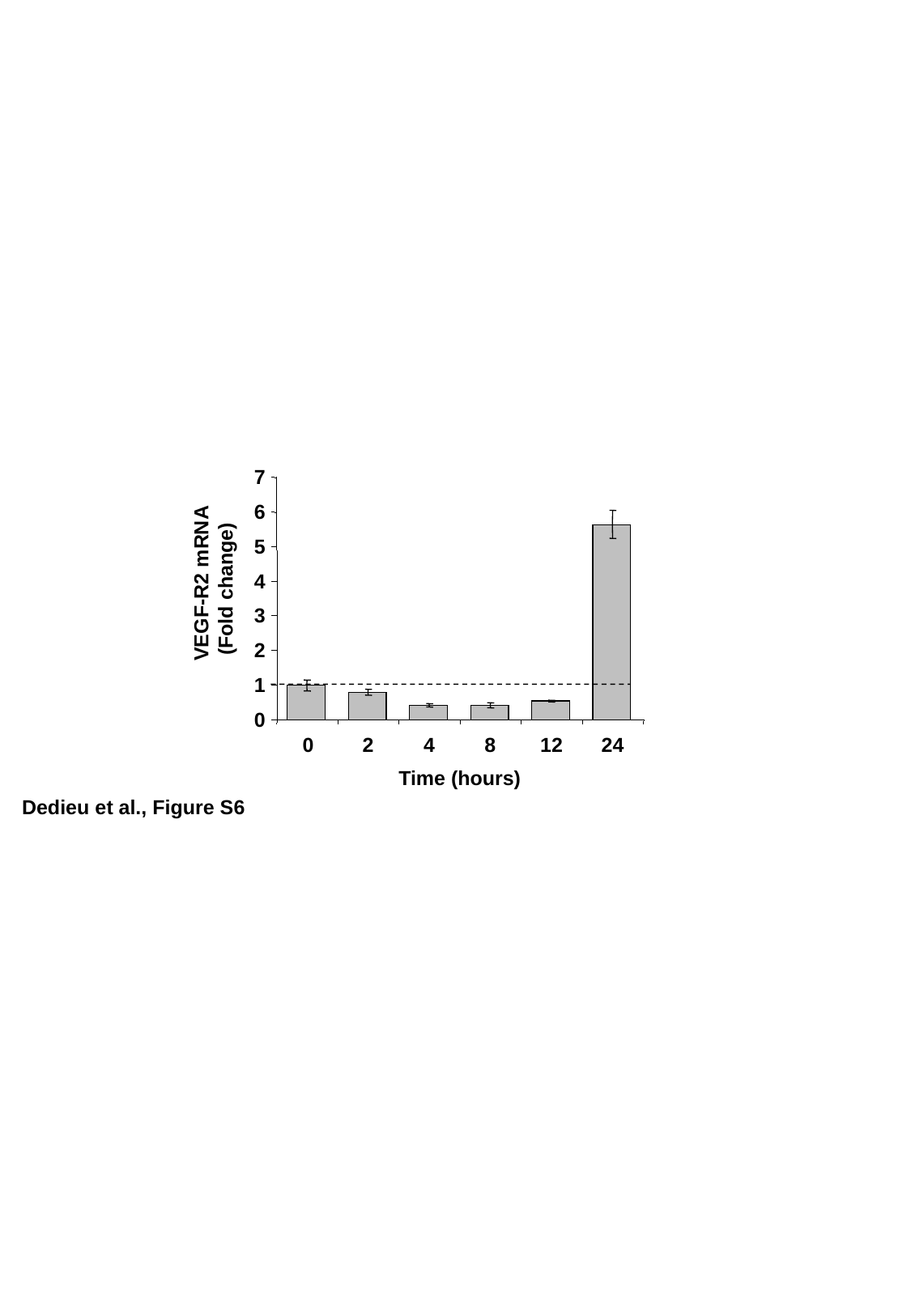

7
6
5
VEGF-R2 mRNA
 (Fold change)
4
3
2
1
0
0
2
4
8
12
24
Time (hours)
Dedieu et al., Figure S6

Supplement: Additional file 5 — Figure S6. Amifostine treatment decreases vascular endothelial growth factor-R2 mRNA expression or stability. After a 24 h-starvation, subconfluent human umbilcal vein endothelial cells were incubated in the presence or absence of 1 mM WR1065 and total mRNA were collected at the indicated time points. Reverse transcription and real time quantitative polymerase chain reaction was performed as previously described (Methods). Results are shown in fold induction as compared to control untreated cells. They were normalized to α-tubulin and correspond to the mean values ± standard deviation of triplicates from three independent experiments. See Additional File 2 for materials and methods for supplementary figures. [file 1741-7015-8-19-S5.PPT]
