# Supplementary material for: The cytoprotective drug amifostine modifies both expression and activity of the pro-angiogenic factor VEGF-A
Source: BMC Med. 2010 Mar 24;8:19. doi: 10.1186/1741-7015-8-19 (PMC2859403; doi:10.1186/1741-7015-8-19)
Supplement: Additional file 4 — Figures S4 and S5. (Figure S4) Amifostine inhibits the proliferation and migration of BAE cells, as well as VEGF-A signalling in those cells. Figure S5. Amifostine inhibits cell cycle progression of BCE in G1. See additional file 2 for materials and methods for supplementary figures. [file 1741-7015-8-19-S4.PDF]

Figure S4 -A. Amifostine inhibits BAE cells proliferation. BAE were inoculated at 7500 cells/cm^2 in 0.2% gelatine-coated 24 multiwell plates. Cells were then allowed to grow for three days in complete culture medium, containing increasing concentrations of amifostine (WR-2721). Cell densities were determined using a cell counter. Each result is expressed as the mean of triplicate determinations ± SEM. The experiment was carried out three times with similar results. Symbols used are as follows : no WR-2721; WR-2721 0.25 mM ; 0.5 mM ; 1 mM ; 2 mM. B. Amifostine inhibits BAE cells migration. Cells were allowed to grow in complete culture medium until forming a confluent monolayer. They were then serum-starved for 24h in DMEM supplemented with 1% L-Glutamine, after which they were scratched over a define zone, and treated with aminoguanidine alone or supplemented with 2 mM amifostine (WR-2721), in the presence or absence of 25 ng/ml VEGF-A. After 14h of migration, cells were fixed and stained with May-Grunwald and Giemsa. The extent of migration in the various experimental settings was assessed by counting cells that invaded the wound zone. Left panel : histogram representation of cell migration in the presence (grey solid bars) or absence (white solid bars) of VEGF-A. Results are expressed as percentages of migrated cells, calculated relatively to the number of cells counted in the control condition (cells

treated with AG only, in the absence of any VEGF-A stimulation). Data are means of at least four counts, performed in randomly chosen fields. Experiments were carried out three times with similar results. Right panel : representative microphotographs of cells after fixation and coloration, taken under an inverted microscope at a 100-fold magnification. C. Amifostine treatment impairs MAPK signaling in BAE cells. The experiment was performed six times with similar results, following the same procedure as for HUVEC (see fig. 7C).

Figure S5 - Bovine Capillary EC (BCE) inoculated at 10000 cells/cm^2 were allowed to grow in complete culture medium supplemented with aminoguanidine and several doses of amifostine (WR-2721), for increasing periods of time. At each time point, starting from time 0, cells were

trypsinized and prepared for cell cycle analysis using the Cycle Test Plus staining kit TM (Becton Dickinson). Cell cycle profiles obtained are shown for untreated (NT) cells, and cells treated with increasing concentrations of WR-2721 (0, 0.5, 1 and 2 mM) in the presence of 2 mM AG. The histogram shows the percentage of cells in each phase of the cycle for the corresponding treatment.

See additional file 2 for materials and methods for supplementary figures.

A.

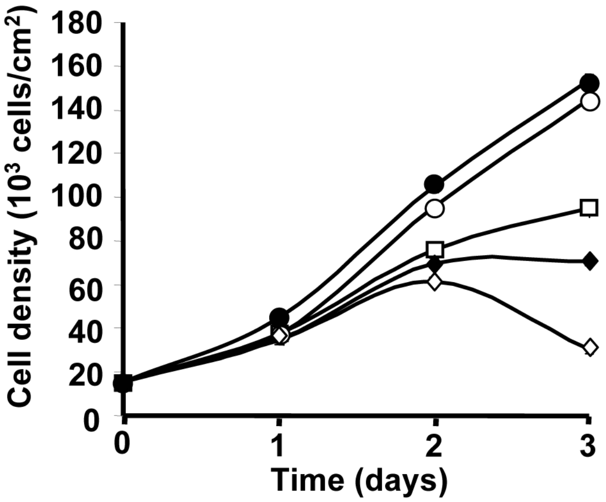

Cell density (10^3 cells/cm^2)
180
160
140
120
100
80
60
40
20
0
0
1
2
3
Time (days)

B.

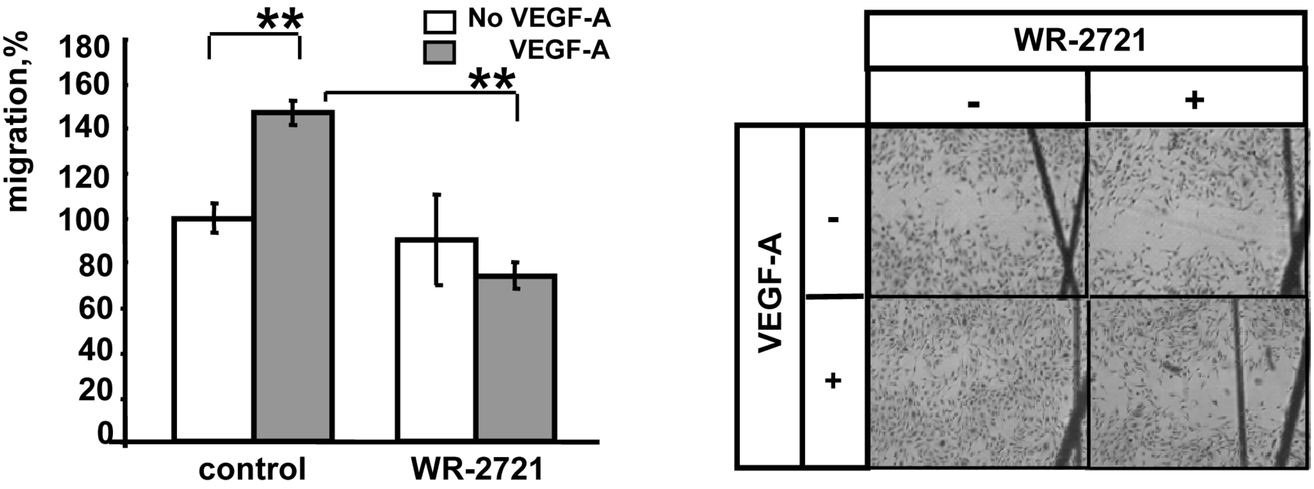

No VEGF-A
VEGF-A
**
**
migration,%
180
160
140
120
100
80
60
40
20
0
control
WR-2721
WR-2721
-
+
VEGF-A
-
+

C.

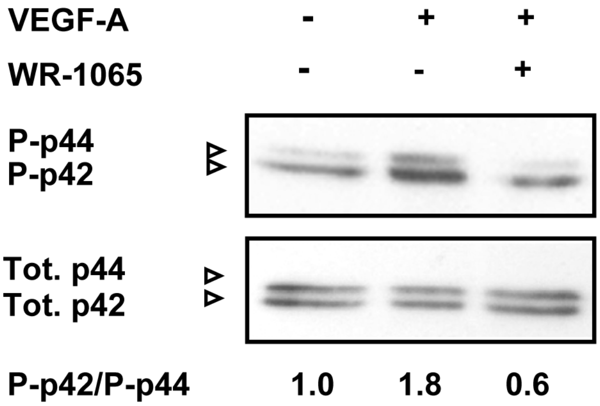

VEGF-A - + +
WR-1065 - - +
P-p44
P-p42
Tot. p44
Tot. p42
P-p42/P-p44 1.0 1.8 0.6

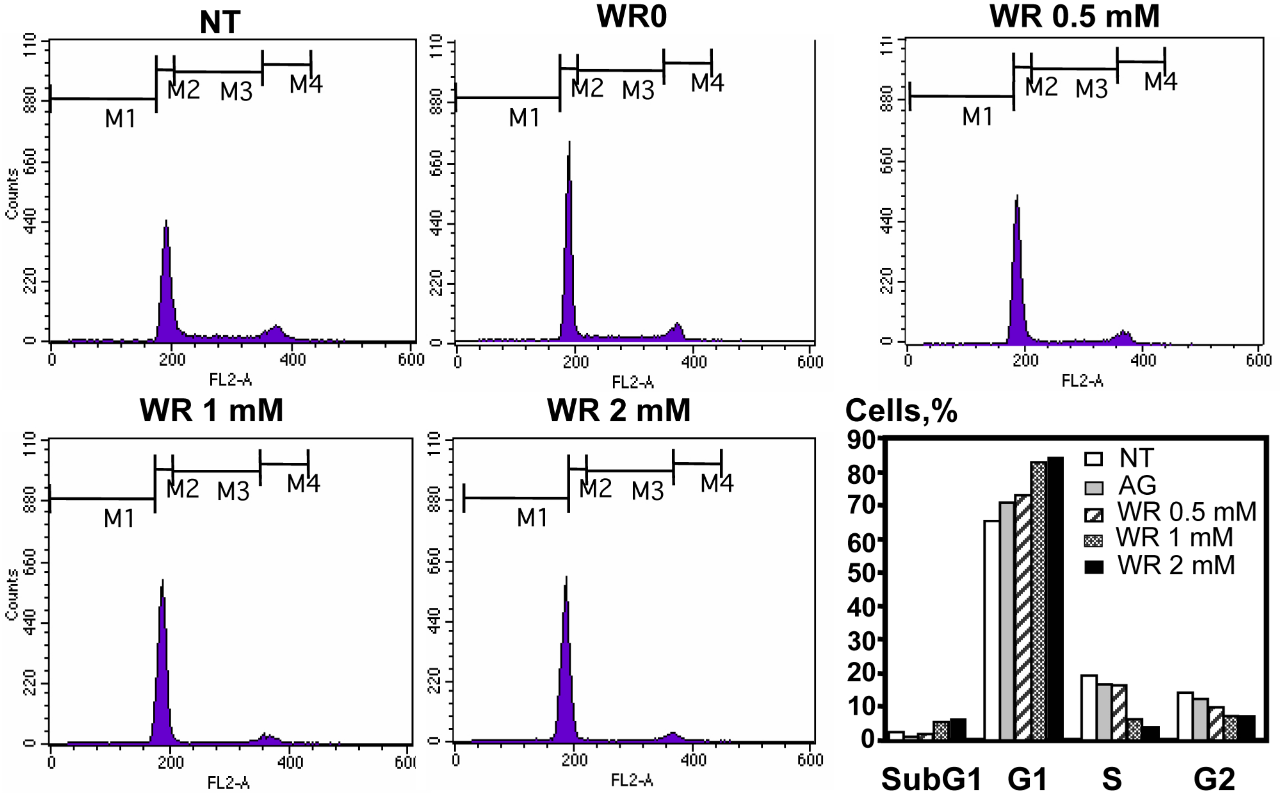

Dedieu et al., FIGURE S5
